# Nucleocapsid protein-dependent assembly of the RNA packaging signal of Middle East respiratory syndrome coronavirus

**DOI:** 10.1186/s12929-018-0449-x

**Published:** 2018-05-24

**Authors:** Wei-Chen Hsin, Chan-Hua Chang, Chi-You Chang, Wei-Hao Peng, Chung-Liang Chien, Ming-Fu Chang, Shin C. Chang

**Affiliations:** 1 Institute of Microbiology, College of Medicine, National Taiwan University, No. 1, Jen-Ai Road, First Section, Taipei, 100 Taiwan; 2 Institute of Anatomy and Cell Biology, College of Medicine, National Taiwan University, No. 1, Jen-Ai Road, First Section, Taipei, 100 Taiwan; 3 Institute of Biochemistry and Molecular Biology, College of Medicine, National Taiwan University, No. 1, Jen-Ai Road, First Section, Taipei, 100 Taiwan

**Keywords:** MERS-CoV, RNA packaging signal, Nucleocapsid protein

## Abstract

**Background:**

Middle East respiratory syndrome coronavirus (MERS-CoV) consists of a positive-sense, single-stranded RNA genome and four structural proteins: the spike, envelope, membrane, and nucleocapsid protein. The assembly of the viral genome into virus particles involves viral structural proteins and is believed to be mediated through recognition of specific sequences and RNA structures of the viral genome.

**Methods and Results:**

A culture system for the production of MERS coronavirus-like particles (MERS VLPs) was determined and established by electron microscopy and the detection of coexpressed viral structural proteins. Using the VLP system, a 258-nucleotide RNA fragment, which spans nucleotides 19,712 to 19,969 of the MERS-CoV genome (designated PS258(19712–19969)_ME_), was identified to function as a packaging signal. Assembly of the RNA packaging signal into MERS VLPs is dependent on the viral nucleocapsid protein. In addition, a 45-nucleotide stable stem-loop substructure of the PS258(19712–19969)_ME_ interacted with both the N-terminal domain and the C-terminal domain of the viral nucleocapsid protein. Furthermore, a functional SARS-CoV RNA packaging signal failed to assemble into the MERS VLPs, which indicated virus-specific assembly of the RNA genome.

**Conclusions:**

A MERS-oV RNA packaging signal was identified by the detection of GFP expression following an incubation of MERS VLPs carrying the heterologous mRNA GFP-PS258(19712–19969)_ME_ with virus permissive Huh7 cells. The MERS VLP system could help us in understanding virus infection and morphogenesis.

## Background

Middle East respiratory syndrome coronavirus (MERS-CoV) is a novel coronavirus that causes acute respiratory syndrome with a high mortality rate in human [1]. The first case of MERS-CoV was identified in September 2012 in Saudi Arabia. From the initial outbreak to April 2018, MERS-CoV spread through 27 countries and caused approximately 2144 cases and 750 deaths (http://www.who.int/emergencies/mers-cov/en/). Dipeptidyl peptidase 4, which is also known as CD26, was identified as a functional receptor for MERS-CoV [2, 3]. Coronaviruses are enveloped, positive-sense, single-stranded RNA viruses with approximately 26–32 kb genomic RNA [4–6]. The 5′ two-thirds of the viral genome consists of open reading frames (ORF) 1a and 1b that encode the viral nonstructural proteins (nsps), whereas the 3′ one-third consists of ORFs that encode the viral structural proteins, including the spike (S), membrane (M), envelope (E) and nucleocapsid (N), and accessory proteins. The S, M and E proteins form the viral envelope [7–9], whereas the N protein is considered the most important protein that interacts with the viral genomic RNA and packages the RNA into virus particles by recognizing a specific sequence, which is termed the packaging signal [10–12].

Studies on mouse hepatitis virus (MHV), which is a lineage A betacoronavirus, localized the packaging signal to the 3’-end of ORF 1b of the viral genome in the gene that encodes the nsp15 endoribonuclease [13–15]. A stable stem-loop of 69-nucleotides (nt) was identified as sufficient for RNA packaging. Computer analysis further revealed conservation of the packaging signal in both the sequence and RNA secondary structure between MHV and bovine coronavirus (BCoV) and among other lineage A betacoronaviruses [16–18]. Our previous studies on severe acute respiratory syndrome coronavirus (SARS-CoV), which is a lineage B betacoronavirus, identified a 580-nt RNA fragment with a stable stem-loop structure as a functional signal to drive packaging of coexpressed RNA of a green fluorescent protein (GFP) into virus-like particles (VLPs) [19]. This SARS-CoV packaging signal spanned the viral genome from nt 19,715 to 20,294 and was localized to a subdomain of the nsp15 gene different from the packaging signal of lineage A betacoronaviruses. However, studies on alphacoronavirus transmissible gastroenteritis virus (TGEV) localized the RNA packaging signal to the 5′-end of the viral genome [20, 21], and sequences in the 5’-UTR and/or 3’-UTR may be required for the RNA packaging of gammacoronavirus infectious bronchitis virus (IBV) [22, 23].

In addition to the packaging signal, the viral structural proteins may also play roles in the assembly of the viral genome. Studies on MHV indicated that M and E proteins are sufficient for the assembly of VLPs [9]. Through a specific interaction between the viral M protein and the packaging signal, a coexpressed RNA fragment carrying the viral packaging signal can be packaged into VLPs in the absence of N protein [24, 25]. Nevertheless, studies argue for the involvement of the N protein in RNA genome packaging. The carboxy-terminal domain (CTD) of the N protein was demonstrated to be the major determinant of MHV packaging signal recognition [11]; and the further downstream carboxy-terminal tail coordinates the selectivity of genome packaging and couples genome encapsidation to the virion assembly [26]. In addition, our earlier study on SARS-CoV indicated N protein-dependent assembly of the viral RNA packaging signal [19]. To date, the RNA packaging signal of MERS-CoV has not been determined, and it is unclear whether the N protein is essential for the MERS-CoV RNA package.

In this study, a putative RNA packaging signal of the MERS-CoV genome was predicted by bioinformatics analysis. MERS VLPs carrying RNA sequences of GFP fused to a 258-nt RNA fragment consisting of the putative RNA packaging signal and its flanking sequences were generated and incubated with MERS-CoV-permissive Huh7 cells for functional analysis. The results indicate that the RNA fragment spanning nt 19,712 to 19,969 of the viral genome is sufficient to function as a packaging signal and assemble into the MERS VLPs. In addition, packaging of the MERS-CoV RNA depends on the viral N protein.

## Methods

### Cell lines and culture condition

Human embryonic kidney cells 293 T and hepatocellular carcinoma Huh7 cells were maintained in Dulbecco’s modified Eagle medium (GIBCO) supplemented with 10% heat-inactivated fetal bovine serum, 1% 100X nonessential amino acids plus 100 U of penicillin and 100 μg of streptomycin per ml in a humidified 5% CO_2_ atmosphere at 37 °C.

### Plasmids

(i) Plasmids pcDNA-ctrl-V5HisTopo, pcDNA-MERS-E, pcDNA-MERS-M, and pcDNA-MERS-N. Plasmid pcDNA-ctrl-V5HisTopo was generated by inserting a DNA fragment containing multiple cloning sites derived from annealing of two oligonucleotides, CACCATGGAATTCATCGATCTAGATCCGCGGCCGCACTCGAGT and its complementary sequences, into pcDNA3.1D/V5-His-TOPO (Invitrogen). Plasmids pcDNA-MERS-E, pcDNA-MERS-M, and pcDNA-MERS-N encode the V5- and His-tagged E, M, and N protein, respectively, of MERS-CoV and were generated by inserting their corresponding cDNA fragments into pcDNA-ctrl-V5HisTopo. The MERS-CoV cDNA fragments were chemically synthesized (Invitrogen) according to GenBank (HCoV-EMC strain; accession number NC_019843.3) and used as templates for PCR amplification with the following primer sets: CS-ME-E1 (GGAATTCATGTTACCCTTTG) and CS-ME-E2 (ATCAACCCACTCGTCAGG) for the E gene, CS-ME-M1 (GGAATTCATGTCTAATATGACGC) and CS-ME-M2 (ATCAGCTCGAAGCAATGCAAG) for the M gene, and CS-ME-N-f(RI) (GGAATTCATGGCATCCCCT) and CS-ME-N-r(RI) (GGAATTCATCAGTGTTAACATC) for the N gene. The PCR products of E and M gene were treated with EcoRI and EcoRV restriction endonucleases, and inserted into the EcoRI and EcoRV sites of pcDNA-ctrl-V5HisTopo to generate plasmids pcDNA-MERS-E and pcDNA-MERS-M, respectively. The PCR product of N gene was treated with EcoRI restriction endonuclease, and inserted into the EcoRI site of pcDNA-ctrl-V5HisTopo to generate plasmid pcDNA-MERS-N.

(ii) Plasmid pcDNA-MERS-S. For construction of plasmid pcDNA-MERS-S, pcDNA-MERS-S1 was first generated following a treatment of plasmid pCR4-MERS-S1-Topo (kindly provided by Haagmans BL and Osterhaus AD, Department of Viroscience, Erasmus Medical Center, Rotterdam, Netherlands) with EcoRI restriction endonuclease and insertion of the resultant DNA fragment containing the MERS-CoV S protein coding sequences from nt 1 to 2238 into the EcoRI site of pcDNA-ctrl-V5HisTopo. A DNA fragment representing the MERS-CoV S protein coding sequences from nt 2230 to 4062 with ScaI site at the 5′ end and EcoRV at the 3′ end was then generated by PCR amplification from a chemically synthesized template (Invitrogen) and inserted into the ScaI site of the plasmid pcDNA-MERS-S1 located at nt 2229 of the MERS-CoV S coding sequences to generate plasmid pcDNA-MERS-S.

(iii) Plasmids pET-MERS-N, pET-MERS-N(1–156), and pET-MERS-N(1–263). Plasmids pET-MERS-N, pET-MERS-N(1–156), and pET-MERS-N(1–263) encode the His-tagged full-length MERS-CoV N protein, the N-terminal domain from amino acid residues 1 to 156, and 1 to 263, respectively. For construction of pET-MERS-N, plasmid pcDNA-MERS-N was treated with EcoRI restriction endonuclease and the resultant DNA fragment of the N gene was inserted into the EcoRI site of pET-28a (Novagen). Plasmid pET-MERS-N(1–156) was derived from pET-MERS-N following a digestion with HindIII restriction endonuclease and self-ligation of the resultant 5.8-kb DNA fragment. For construction of plasmid pET-MERS-N(1–263), a 321-bp HindIII fragment was obtained from pET-MERS-N and inserted into the HindIII site of pET-MERS-N(1–156).

(iv) Plasmids pET-MERS-N(239–413) and pET-MERS-N(264–413). Plasmids pET-MERS-N(239–413) and pET-MERS-N(264–413) encode the His-tagged MERS-CoV N protein from amino acid residues 239 to 413 and 264 to 413, respectively. For generation of plasmid pET-MERS-N(239–413), pET-MERS-N was used as the template for PCR amplification with the primer set CS-ME-N715(NheI)-f (GGGGCTAGCACTAAGAAAGATGCTGC) and CS-ME-N-r(RI) (GGAATTCATCAGTGTTAACATC). The resultant PCR product representing the DNA fragment of N(239–413) was treated with NheI and EcoRI restriction endonucleases and then inserted into the NheI and EcoRI sites of pET-28a. For generation of plasmid pET-MERS-N(264–413), a 468-bp DNA fragment obtained from the treatment of pET-MERS-N with HindIII restriction endonuclease was inserted into the HindIII site of pET-28a.

(v) Plasmids pEGFP-PS258(19712–19969)ME and pEGFP-N1-PS580. Plasmid pEGFP-PS258(19712–19969)ME represents the cDNA of the putative packaging signal of MERS-CoV genomic RNA inserted into the 3′ noncoding region of the GFP gene. For construction of the plasmid pEGFP-PS258(19712–19969)ME, a DNA fragment representing MERS-CoV genomic RNA from nt 19,712 to 19,969 with NotI site on both ends was first obtained following PCR amplification from a chemically synthesized template (Invitrogen) and a treatment of the PCR product with NotI restriction endonuclease. The DNA fragment was then inserted into the NotI site of pEGFP-N1 to generate plasmid pEGFP-PS258(19712–19969)ME. Plasmid pEGFP-N1-PS580 has been previously described [19]. It represents the cDNA of the packaging signal of SARS-CoV genomic RNA from nt 19,715 to 20,294 inserted into the 3′ noncoding region of the GFP gene.

### Transient transfection and harvest of cell lysates

Transient transfection was performed with T-Pro Non-liposome transfection Reagent II (Genestar Biotech) according to the manufacturer’s protocol. Briefly, the expression plasmid was diluted into Opti-MEM (GIBCO) and mixed with T-Pro Non-liposome transfection Reagent II at the ratio of 1 μg DNA to 2 μl transfection Reagent. Two days posttransfection, cells were lysed using a RIPA buffer consisting of 50 mM Tris-HCl, pH 7.5, 150 mM NaCl, 0.5% sodium deoxycholate, 1% NP-40, 0.1% SDS, and 1% complete EDTA-free protease inhibitor (Roche).

### Production and harvest of virus like particles (VLPs)

For production of MERS VLPs, cotransfection was performed with plasmids encoding the MERS-CoV structural proteins E, M, and S, in the presence or absence of the N-expressing plasmid and the MERS-CoV packaging signal plasmid pEGFP-PS258(19712–19969)ME, the SARS-CoV packaging signal plasmid pEGFP-N1-PS580, or the vector control pEGFP-N1. Production of MERS VLPs in the transfected cells was examined by electron microscopy three days posttransfection. For the collection of supernatant VLPs, culture medium was clarified by centrifugation at 1000 rpm in an RS-240 rotor (Kubota 2010) for 5 min, passed through a 0.45 μm filter, and subjected to ultracentrifugation at 36000 rpm in an SW41 rotor (Beckman) for 3 h at 4 °C. The VLP pellet was resuspended in PBS and stored at − 80 °C. For collecting cellular VLPs, the transfected cells were resuspended in PBS containing 1% complete EDTA-free protease inhibitor and subjected to three cycles of freeze (− 80 °C) and thaw (37 °C) prior to the centrifugation at 1000 rpm. In addition, for separation of supernatant VLPs with different structure compositions, the VLP suspension was further loaded on a discontinuous sucrose gradient consisting of 20, 30, 50, and 60% sucrose in 20 mM HEPES (pH 7.4) and 0.1% BSA and centrifuged at 36000 rpm for 15 h at 4 °C. The density of VLPs was determined by a refractometer (ATAGO). Expression of MERS-CoV structural proteins in the VLPs were examined by Western blot analysis.

### Electron microscopic analysis of MERS VLPs

Three days following cotransfection of the plasmids encoding the MERS-CoV structural proteins, cells were fixed with 4% glutaraldehyde in 0.1 M phosphate buffer for 24 h at 4 °C and post-fixed 1% aqueous osmium tetroxide diluted in the same phosphate buffer at room temperature for 1 h. After washing, the fixed cells were dehydrated in a graded ethanol series and embedded in a Polybed 812-Araldite mixture (EMS, Hatfield, PA). Semi-thin sections of 1 μm were obtained using an ultramicrotome (Ultracut E, Leica-Reichert Jung, Wetzlar, Germany) and stained with toluidine blue for correlative light microscopy. Ultrathin sections were cut at 70 nm, collected on copper grids (200 meshes) and stained with uranyl acetate and lead citrate. Images were examined in a Hitachi H-7100 electron microscope equipped with a Gatan 832 digital camera (Gatan, Inc.).

### Western blot analysis

Protein lysates were resolved by polyacrylamide gel electrophoresis (PAGE) and electrotransferred onto an Immobilon-P membrane (Millipore) as described previously [27]. Mouse monoclonal antibody against V5 epitope (Invitrogen) was used as the primary antibody to detect expression of the V5-tagged recombinant proteins. Following incubation with horseradish peroxidase-conjugated secondary antibodies, specific interactions between antigens and antibodies were detected by the enhanced chemiluminescence system (Advansta).

### Immunofluorescence assay

Cells cultured on glass coverslips were fixed with 4% paraformaldehyde for 30 min at room temperature. The fixed cells were permeabilized with 0.5% Triton X-100 and washed with PBS. Following a blocking with 3% BSA for 1 h at room temperature, the cells were incubated with rabbit polyclonal antibodies against GFP for 1 h at room temperature, washed with PBS, and then incubated with secondary antibodies conjugated with Alexa Fluor® 488 (Invitrogen) for 1 h at room temperature. Cell nuclei were stained with Hoechst. The coverslips were mounted on glass slides. Zeiss Axioskop 40 microscope was used to capture the images.

### Expression and purification of MERS-CoV N protein and its subdomains

For expression of recombinant MERS-CoV N protein and its subdomains, plasmids pET-MERS-N and its derivatives were individually transformed into *E. coli* BL21(DE3) cells. Protein expression was induced with 0.1 mM isopropyl-β-D-thiogalactopyranoside for 18 h at 16 °C, and purified as previously described [19] with modifications. In brief, cell pellets were subjected to sonication and centrifugation at 6000 rpm in an RA-200 J rotor (Kubota) for 10 min at 4 °C. The supernatants were collected and loaded onto a nickel affinity column. The His-tagged MERS-CoV N proteins were eluted with elution buffers consisting of 50 mM sodium phosphate, pH 8.0, 300 mM NaCl, 6 M urea and a stepwise gradient of imidazole at 10, 50 and 200 mM. The fractions that contain proteins of interest, identified by Coomassie blue staining and Western blot analysis following SDS-PAGE, were pooled together and dialyzed against a dialysis buffer containing 50 mM sodium phosphate, pH 7.4, 150 mM NaCl, 1 mM EDTA, and 0.01% NaN_3_. The purified proteins were concentrated with Amicon Ultra-15 concentrator (Millipore) and kept in 50% glycerol at − 80 °C for filter binding assay.

### Filter binding assay

Filter binding assay was carried out as previously described [19] with modifications. In brief, biotinylated RNA fragments were biochemically synthesized (Thermo Fisher; Integrated DNA Technologies) and used as the probes. The probes were heated at 80 °C for 10 min and then incubated at 37 °C for 15 min. Purified proteins pre-incubated independently at 37 °C for 15 min were then mixed with the RNA probe in a filter binding buffer consisting of 20 mM HEPES, pH 7.3, 40 mM KCl, 2 mM MgCl_2_, and 2 mM DTT. After an additional incubation for 10 min, the reaction mixture was passed through Immobilon-P membrane that had been activated with methanol and soaked in the filter binding buffer. The membrane was washed three times with the binding buffer. Specific interactions between biotin-labeled RNA probes and purified proteins were detected by the enhanced chemiluminescence system following pre-incubation with streptavidin horseradish peroxidase conjugate. In addition, a biotin-labeled RNA fragment 5′–UCCUGCUUCAACAGUGCUUGGACGGAAC–3′ (Thermo Fisher) was used as the negative control.

## Results

### Bioinformatics analysis of the MERS-CoV RNA genome

**Fig. 1:**
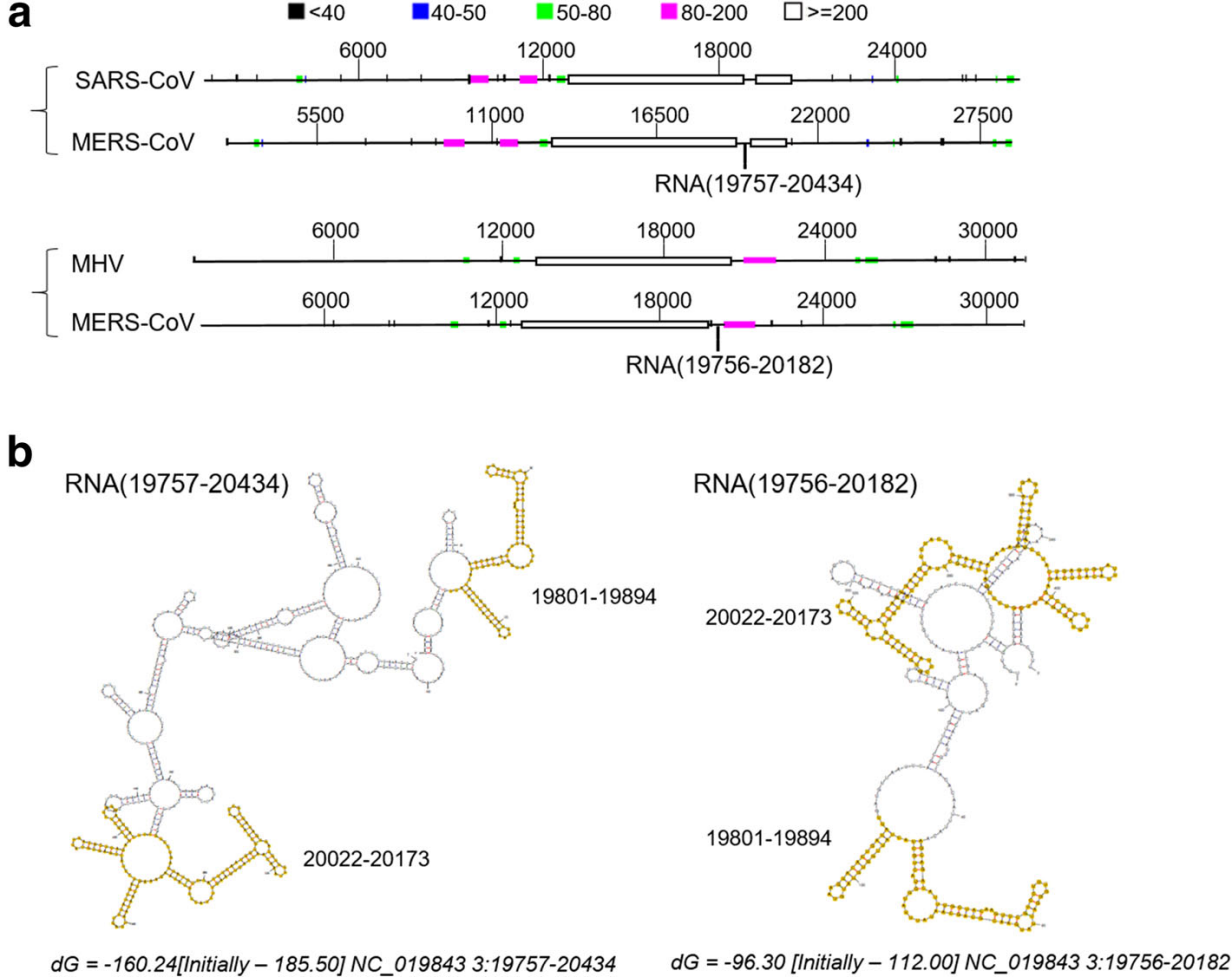
Sequence similarity among the coronavirus genomic RNAs and secondary structure prediction. **a** Alignment of the whole genome sequence of MERS-CoV (accession number NC_019843.3) with that of SARS-CoV (accession number AY291451.1) and MHV (accession number NC_001846.1) by NCBI blastn. Regions with the highest conservation (alignment score ≧ 200) are indicated by open boxes followed by alignment scores 80–200, 50–80, 40–50, and < 40 as indicated. MERS-CoV RNA fragments spanning nt 19,757 to 20,434 [RNA(19757–20434)] and nt 19,756 to 20,182 [RNA(19756–20182)] with sequence diversity were subjected to secondary structure prediction. **b** Secondary structure prediction. Secondary structure of MERS-CoV RNA(19757–20434) and RNA(19756–20182) were analyzed by Mfold. Two subdomains of 94-nt (nt 19,801 to 19,894) and 152-nt (nt 20,022 to 20,173) that form stable substructures in both RNA fragments were colored yellow

Bioinformatics and functional analysis have previously identified the minimum size packaging signal (approximately 60 to 95 nt) for various coronaviruses. However, the packaging signal of lineage C betacoronavirus MERS-CoV has not been determined. Based on the observations that a stable stem–loop structure is often a prerequisite for an RNA fragment to be a packaging signal and that unique sequences may determine the packaging specificity of different coronavirus lineages, we proposed the analysis of the genome sequence and the structure of different lineage betacoronaviruses for the prediction of the potential packaging signal of MERS-CoV. The whole genome sequence of MERS-CoV was first compared with lineage B betacoronavirus SARS-CoV by alignment with NCBI blastn. This revealed a highly variable region from nt 19,757 to 20,434 of the MERS-CoV genome located at the 3′ end of ORF 1b and between two regions that have the highest conservation (the alignment score was higher than 200; Fig. 1a, top). Interestingly, this variable region overlaps the PS580 RNA fragment spanning nt 19,715 to 20,294 of the SARS-CoV genome, which has been identified as a functional packaging signal [19]. In addition, when the MERS-CoV RNA sequence was compared with that of the lineage A betacoronavirus MHV, the divergent sequence was localized to a similar but shorter fragment spanning nt 19,756 to 20,182 of the MERS-CoV genome (Fig. 1a, bottom). These results located a highly variable region of the genome sequences among the different lineage betacoronaviruses. Secondary structures of the MERS-CoV RNA(19757–20434) and RNA(19756–20182) were then analyzed (Fig. 1b). Two RNA subdomains of 94-nt (nt 19,801 to 19,894) and 152-nt (nt 20,022 to 20,173) that form stable structures in both RNA fragments were identified as potential packaging signals of MERS-CoV.

### Identification of PS258(19712–19969)_ME_as a putative N protein-dependent RNA packaging signal of MERS-CoV

**Fig. 2:**
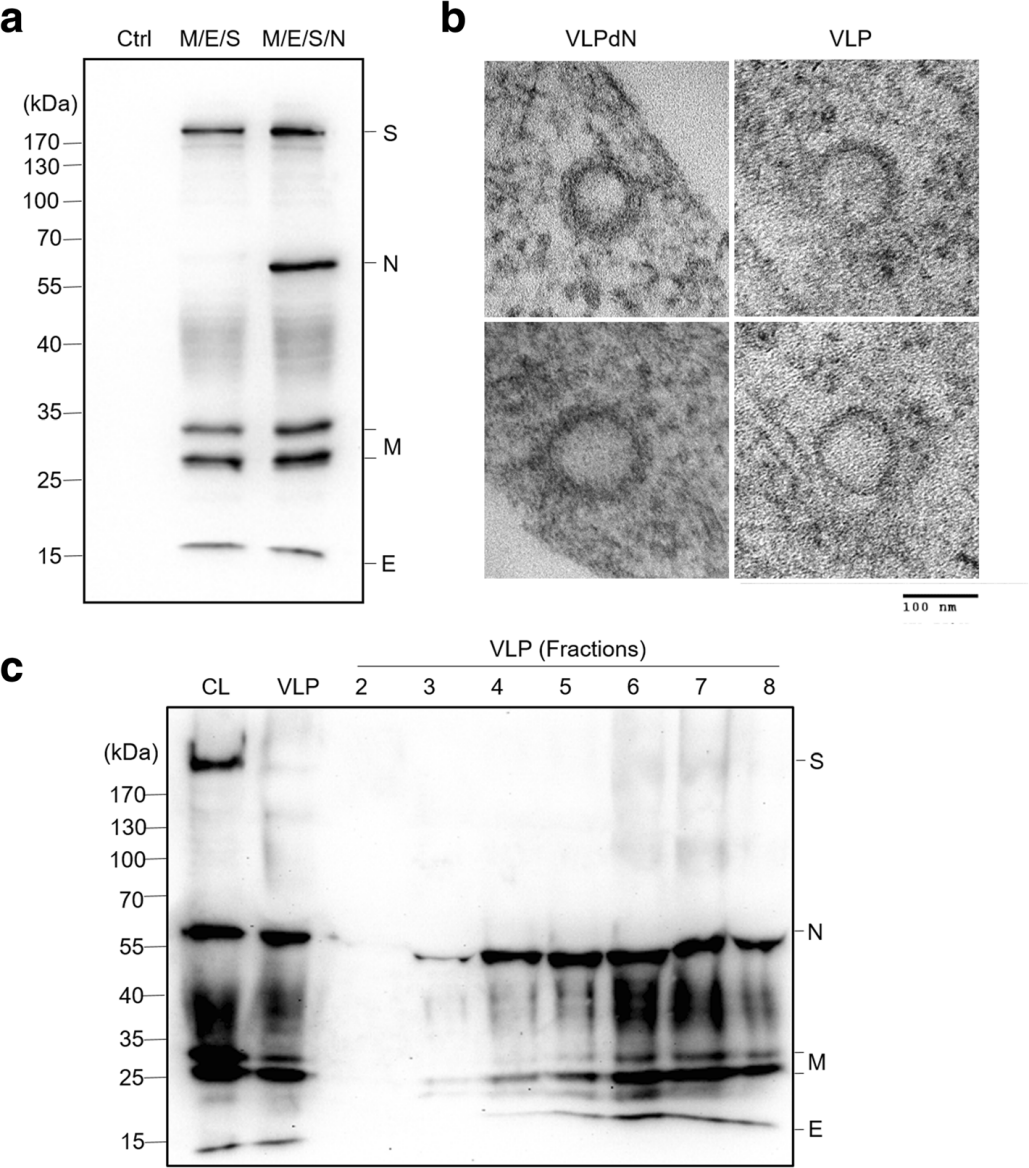
Production of MERS VLPs in cultured cells. **a** Expression of MERS-CoV structural proteins. Plasmids encoding MERS-CoV structural proteins M, E, S, and N as indicated were cotransfected into 293 T cells and the cell lysates were prepared for Western blot analysis two days posttransfection. Protein lysate prepared from cells transfected with control plasmid was applied as a negative control (lane Ctrl). **b** Electron microscopic analysis of MERS VLPs. The cells were fixed and embedded three days following cotransfection of the plasmids encoding the MERS-CoV structural proteins M, E, and S (VLPdN) or M, E, S, and N (VLP). Images of the sections were examined for the presence of MERS VLPs in a Hitachi H-7100 electron microscope equipped with a Gatan 832 digital camera. **c** Separation of MERS VLPs. MERS VLPs collected from culture medium were subjected to a discontinuous sucrose gradient centrifugation as described in Methods. The fractions were collected for Western blot analysis. Lane CL represents the protein lysates of transfected cells and lane VLP represents the total VLPs prior to the gradient centrifugation

For examination of the RNA packaging of MERS-CoV, a culture system that expresses viral structural proteins and produces MERS VLPs was established. As shown in Fig. 2a, expression of MERS-CoV structural proteins was detected following cotransfection of plasmids encoding the viral proteins M, E, S, and N as indicated. Successful production of MERS VLPs in transfected cells in the absence (VLPdN) or presence of N protein (VLP) was confirmed by electron microscopy (Fig. 2b). In addition, VLPs that were released into the culture medium (supernatant VLPs) were subjected to a discontinuous sucrose gradient centrifugation. Western blot analysis demonstrated that fractions 6 and 7 likely represent VLPs that contain four structural proteins with densities of 1.151 and 1.207, respectively (Fig. 2c).

**Fig. 3:**
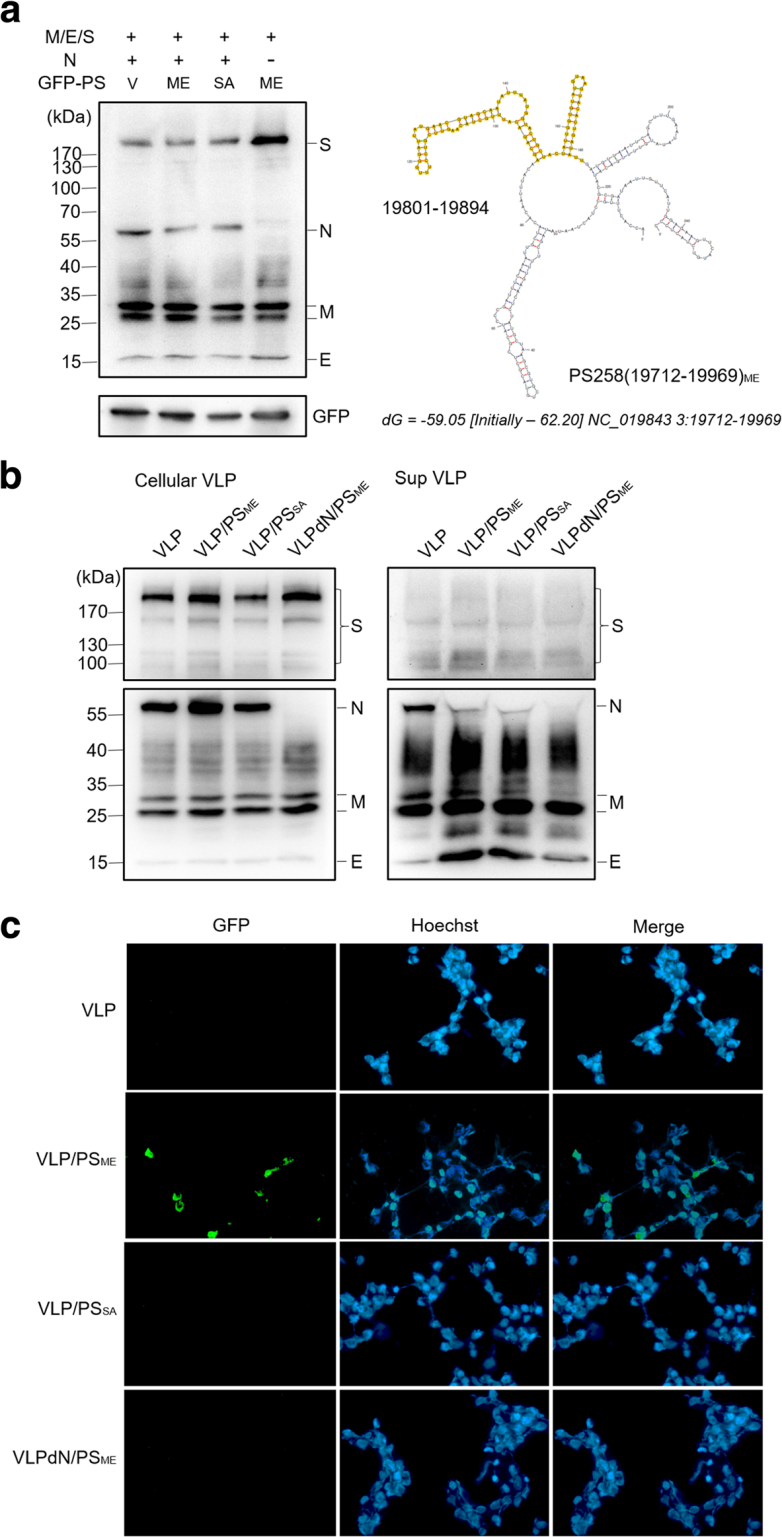
Functional analysis of the putative MERS-CoV RNA packaging signal. **a**-**b** Expression of MERS-CoV structural proteins in transfected cells and VLPs. Western blot analysis was performed with the cell lysates (panel **a**) and both the cellular and supernatant VLPs (panel b) following cotransfection of the plasmids encoding the viral structural proteins and a heterologous GFP-PS mRNA as indicated. The secondary structure of the PS258(19712–19969)_ME_ RNA fragment is shown with the 94-nt stable substructure highlighted as marked in Fig. 1b. **c** Functional analysis of RNA packaging signal. MERS VLPs were collected from the medium of the cultured cells following cotransfection of the plasmids encoding the viral structural proteins S, E, M, and N, and the GFP vector control plasmid pEGFP-N1 (indicated by VLP), the putative MERS-CoV packaging signal plasmid pEGFP-PS258(19712–19969)ME (indicated by VLP/PS_ME_), and the SARS-CoV packaging signal plasmid pEGFP-N1-PS580 (indicated by VLP/PS_SA_). VLPdN/PS_ME_ represents MERSVLPs produced by cotransfection of the plasmids encoding MERS-CoV M, E, and S proteins and plasmid pEGFP-PS258(19712–19969)ME. These MERS VLPs harvested from different cell sources were incubated with naïve Huh7 cells for 48 h. The treated Huh7 cells were then fixed for immunofluorescence staining with GFP antibody and Hoechst

A 258-nt RNA fragment, named PS258(19712–19969)_ME_ (Fig. 3a) and that contains the earlier-identified 94-nt stable stem-loop structure (highlighted) as a center domain and extends on both ends, was chosen to begin functional analysis for identification of the putative RNA packaging signal. To examine the packaging activity of the PS258(19712–19969)_ME_ RNA fragment, the plasmid pEGFP-PS258(19712–19969)ME that expresses heterologous mRNA GFP-PS258(19712–19969)_ME_ was generated. Cotransfection was performed with the plasmids encoding MERS-CoV structural proteins and plasmid pEGFP-PS258(19712–19969)ME. In addition, plasmid pEGFP-N-PS580 that encodes the GFP-PS580 RNA fragment containing the SARS-CoV RNA packaging signal was applied to examine the specificity of the viral RNA packaging. As shown in Fig. 3, the expression of the viral structural proteins was detected in cell lysates (panel a) and in both the cellular and supernatant VLPs (panel b; VLP, VLP/PS_ME_, and VLP/PS_SA_). Assembly of the GFP-PS258(19712–19969)_ME_ into MERS VLPs (VLP/PS_ME_) was examined by incubating the VLPs with the virus-permissible Huh7 cells that endogenously express the viral receptor hDPP4 [2]. As shown in Fig. 3c, GFP-PS258(19712–19969)_ME_ RNA that assembled into the VLP/PS_ME_ was evident by the detection of GFP expression upon entering the cells. The expression of GFP was not detected in Huh7 cells coincubating with VLPs collected from the culture medium of cells cotransfected with plasmids encoding the MERS-CoV structural proteins and the control vector pEGFP-N1 (Fig. 3c; VLP). These results demonstrated that the PS258(19712–19969)_ME_ RNA fragment bears a functional packaging signal that is critical for the assembly of the viral RNA into the MERS VLPs. In the absence of the MERS-CoV RNA sequences, GFP RNA alone was not assembled or expressed in the VLPs. In addition, no GFP expression was detected when the Huh7 cells were incubated with VLPs collected from the culture medium of cells cotransfected with plasmids encoding MERS-CoV structural proteins and the plasmid that encodes the heterologous mRNA GFP-PS580 containing the SARS-CoV RNA packaging signal (Fig. 3c; VLP/PS_SA_), which indicated that the SARS-CoV RNA packaging signal could not be assembled into MERS VLPs. This suggests a viral RNA-specific package.

In addition to the packaging signal, viral structural proteins may also play roles in the assembly of the viral genome [9, 11, 19, 24–26]. To elucidate the MERS-CoV RNA packaging, MERS VLPs produced in the absence of the N protein (named VLPdN/PS_ME_) were incubated with Huh7 cells. In contrast to the expression of GFP in cells incubated with MERS VLP/PS_ME_, the GFP signal was not detected in Huh7 cells incubated with VLPdN/PS_ME_ (Fig. 3c). This result indicates that the MERS-CoV RNA packaging is dependent on the viral N protein.

### MERS-CoV N protein interacts with a stable stem-loop structure in the viral RNA packaging signal

**Fig. 4:**
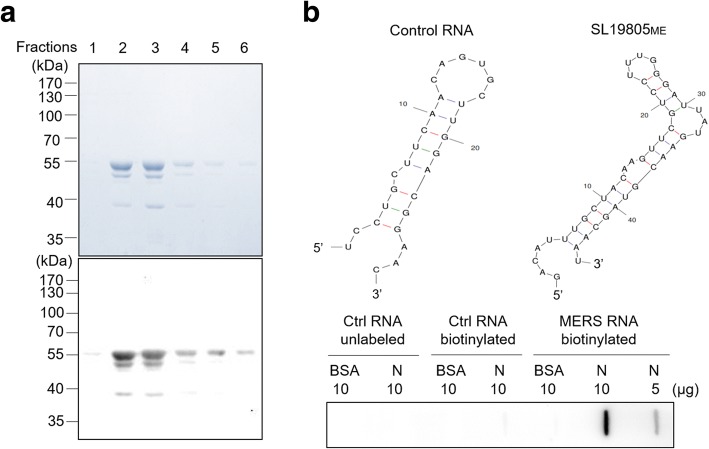
The RNA binding activity of MERS-CoV N protein. **a** Purification of the His-tagged MERS-CoV full-length N protein. Following expression of the His-tagged MERS-CoV N protein in *E. coli* BL21(DE3), the cells were lysed and cell supernatant collected was subjected to protein purification on a nickel-bead affinity column. The His-tagged MERS-CoV N protein eluted by a buffer containing 200 mM imidazole was detected by Coomassie blue staining (top) and Western blot analysis using antibody against the His-tag (bottom). Fractions 2 and 3 were pooled together for the filter binding assay. **b** Filter binding assay. The interactions between the MERS-CoV N protein and the biotin-SL19805_ME_ probe were analyzed by the filter binding assay. Unlabeled and 3’ biotin-labeled RNA fragments with sequences 5′–UCCUGCUUCAACAGUGCUUGGACGGAAC–3′ and the predicted structure (as shown) were used as controls for RNA specificity. BSA was used as a protein control

To investigate the RNA-binding activity of the MERS-CoV N protein, the His-tagged N protein was expressed in *E. coli* BL21(DE3) and purified through a nickel affinity column. As shown in Fig. 4, the His-tagged full-length N protein of approximately 55 kDa was purified by an elution buffer containing 200 mM imidazole (panel a). Fractions 2 and 3, which showed the peak level of the N protein, were pooled together for the filter binding assay. In addition, a 45-nt RNA fragment spanning nt 19,805 to 19,849 (tentatively named SL19805_ME_) of the viral genome that constitutes a stable stem-loop substructure of the PS258(19712–19969)_ME_ was used in the RNA binding assay. As shown in Fig. 4b, an interaction between the biotinylated SL19805_ME_ and the MERS-CoV N protein was detected. However, no signals were observed with the protein control BSA and in the control groups of unlabeled and biotin-labeled RNA. These results localized a predicted substructure of the PS258(19712–19969)_ME_ RNA packaging signal to interact with the viral N protein.

**Fig. 5:**
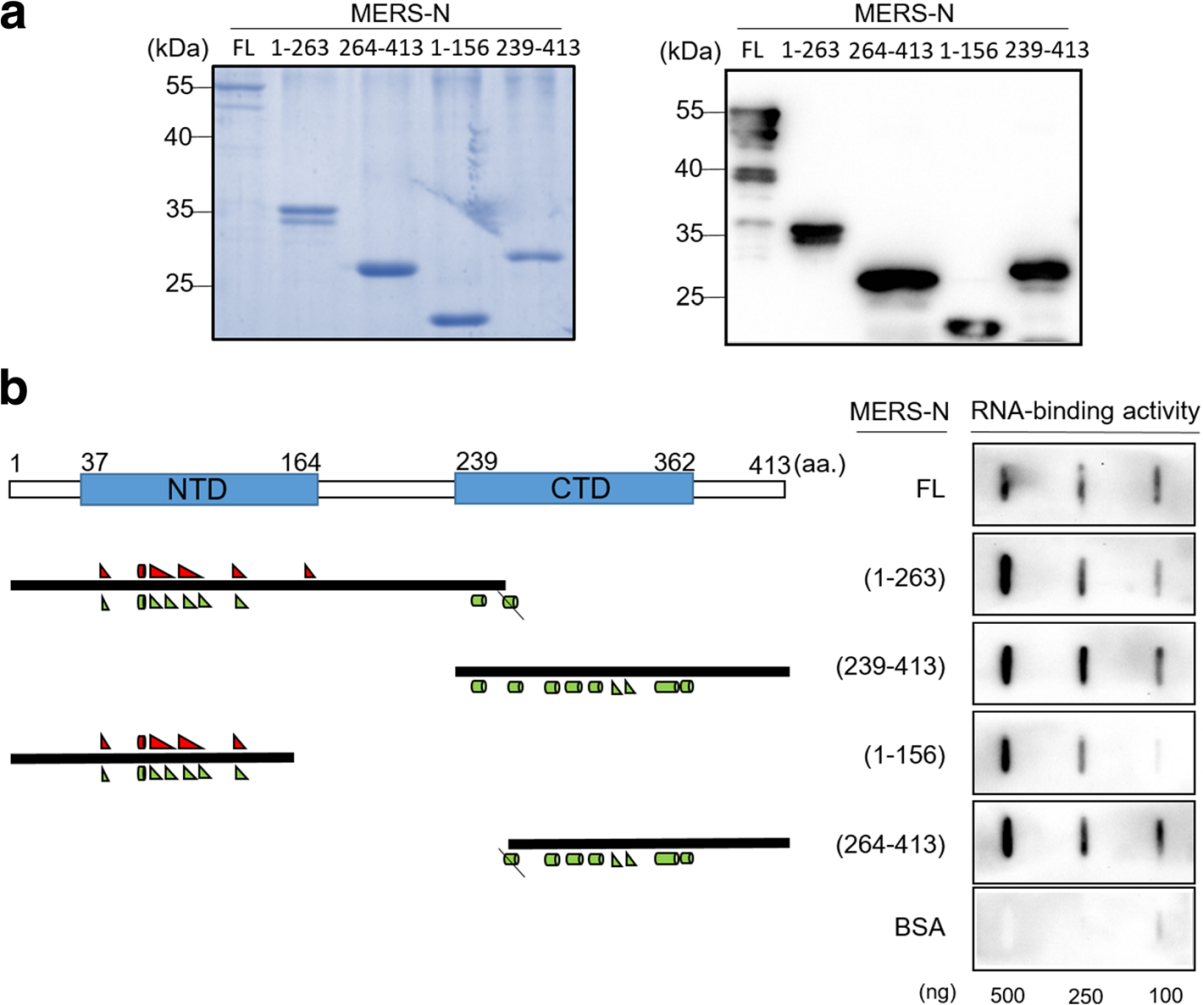
The RNA binding activity of MERS-CoV N fragments. **a** Purification of various N fragments. Purification of the full-length (FL) N protein and its subdomains N(1–263), N(264–413), N(1–156), and N(239–413) followed the procedures as described in the legend of Fig. 4. Coomassie blue staining and Western blot analysis are shown. **b** Schematic representation of the N subdomains and the filter binding assay. The N protein of coronavirus is organized into two structural domains (NTD and CTD) separated by a flexible linker. The NTD structure mainly has an antiparallel β-sheet core domain, whereas the CTD is composed of mainly α-helices [29, 30, 33]. The secondary structural elements are indicated by triangles for β-sheets and cylinders for α-helices, and those shown above the N protein fragments (closed boxes) correspond to the structure of the MERS-CoV N protein, whereas those shown under the boxes correspond to the SARS-CoV N protein. The filter binding assay was conducted with the biotin-SL19805_ME_ and increasing amounts of the N proteins and BSA control as indicated

Although the structure of the full-length MERS-CoV N protein has not been determined, a recent study revealed structural features of the N-terminal region of MERS-CoV in common with SARS-CoV and other coronaviruses [28–30]. The N protein of coronavirus is structurally organized into two domains, the N-terminal domain (NTD) and C-terminal domain (CTD), which are separated by a flexible linker (Fig. 5). The NTD structure is organized as an antiparallel β-sheet core domain with disordered loops, whereas the CTD is composed of mainly α-helices. Previous studies demonstrated that the CTD of the SARS-CoV N protein is involved in oligomerization to form a capsid structure, whereas both domains may be involved in RNA binding [19, 30–34]. In addition, the linker is able to modulate the RNA binding activity of the NTD and CTD [35]. In this study, the RNA-binding characteristics of the MERS-CoV N protein were examined by analyzing the interactions between the SL19805_ME_ RNA and various fragments of the N protein. Following expression and purification of the His-tagged N proteins (Fig. 5a), a filter binding assay was performed. The results demonstrated the RNA binding activities of the full-length N protein and both the N(1–263) and N(239–413) fragments that represent the NTD plus the flexible linker and the CTD, respectively (Fig. 5b). Nevertheless, when deleting a part of the β-sheet structure of the NTD, the N(1–156) fragment had a lower RNA-binding activity compared to that of the N(1–263) fragment, which indicated that the structural integrity of the NTD could be important for RNA binding. However, a 25-amino-acid N-terminal deletion from the N(239–413) fragment, which generated the N(264–413) fragment, had little effect on the RNA-binding activity. These results indicate that both the NTD and CTD of the MERS-CoV N protein could interact with SL19805_ME_, which is a 45-nt stable stem-loop structure in the viral RNA packaging signal.

**Fig. 6:**
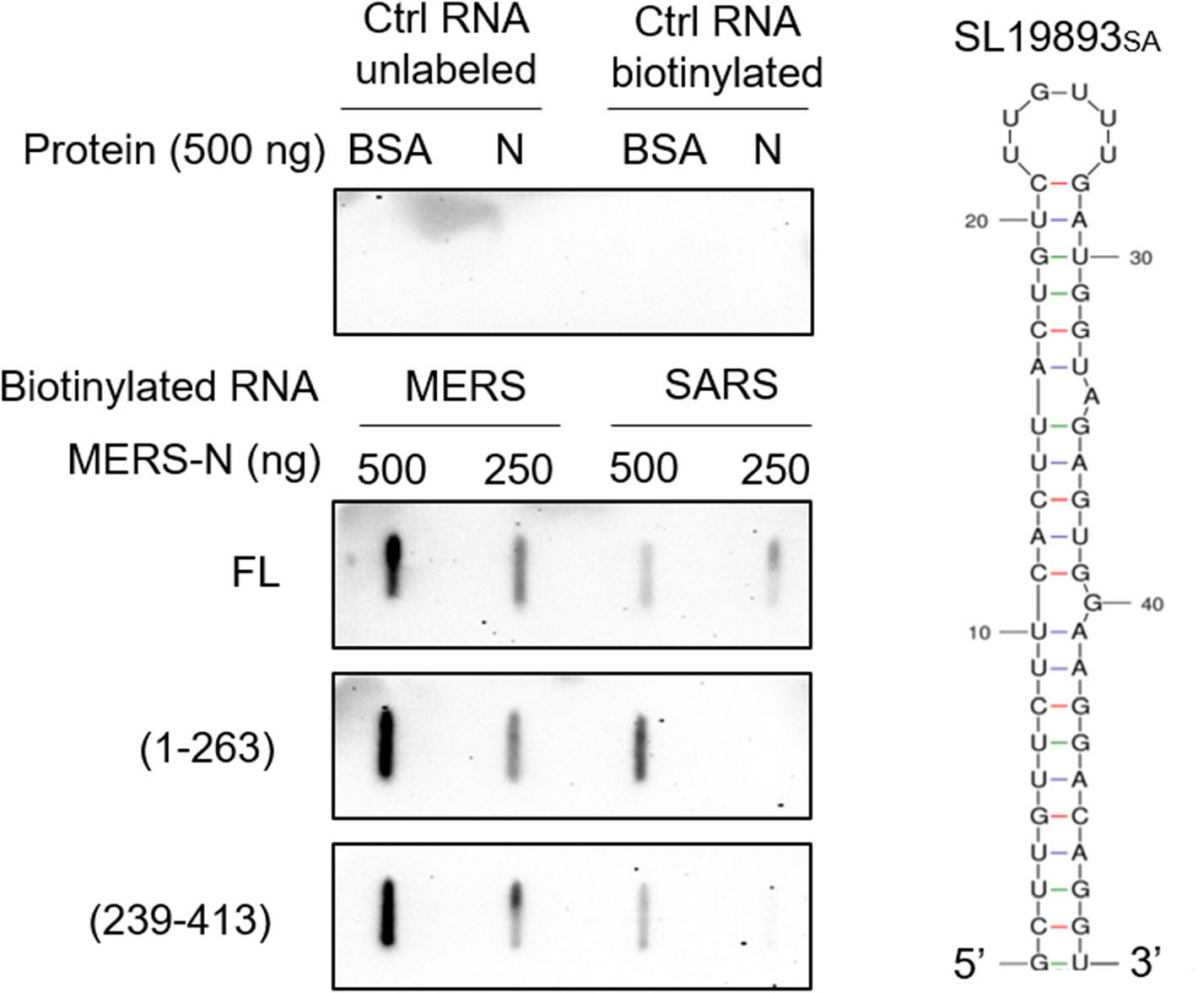
The RNA binding activity of the MERS-CoV N protein on SL19805_ME_ and SL19893_SA_. A filter binding assay was performed with MERS-CoV N proteins and the 50-nt biotin-labeled SARS-CoV RNA (SL19893_SA_). The control RNAs and proteins are described in the legend of Fig. 4

Furthermore, although the heterologous mRNA GFP-PS580 containing the SARS-CoV RNA packaging signal could not be assembled into MERS VLPs (Fig. 3c; VLP/PS_SA_), the SL19893_SA_ that represents a 50-nt conserved stem-loop structure within the PS580 showed its ability to interact with the MERS-CoV N protein (Fig. 6). Nevertheless, the binding activity was much lower than that of the SL19805_ME_. This result suggests that interaction of the viral RNA with the N protein may be a prerequisite but not sufficient for RNA packaging into VLPs. Virus-specific RNA sequences and structures may determine the binding activity of the N protein. In addition, subsequent interactions between the N protein and other viral structural proteins may facilitate assembly of the viral RNA into VLPs.

## Discussion

Our previous study on SARS-CoV indicated an N protein-dependent package of the viral RNA. The RNA packaging signal was biologically identified and located within the viral genome spanning nt 19,715 to 20,294 [19]. In this study, the prediction from the sequence and structure analysis localized the putative MERS-CoV packaging signal to the 3’ end of the ORF 1b of the viral genome (Fig. 1). Further studies demonstrated that the 258-nt PS258(19712–19969)_ME_ RNA fragment could function as a packaging signal and drive the packaging of the heterologous GFP gene into the MERS VLPs (Fig. 3c). Minimum sequences and structures required for RNA packaging need to be further elucidated. Nevertheless, the in vitro RNA binding assay demonstrated an interaction between the viral N protein and a 45-nt stable stem-loop structure within the functionally identified packaging signal of the MERS-CoV RNA (Figs. 4 and 5).

Previously, studies on MHV mapped a 69-nt stem-loop RNA structure as the core packaging signal of MHV, and a 190-nt RNA fragment containing the core packaging signal has a much higher packaging activity [13, 25]. In addition, the packaging signal selectively interacts with the viral M protein, which drives the packaging of the viral RNA into the virus particles [25]. Studies on various coronaviruses indicate that coronaviruses could package the RNA fragments containing their own packaging signals [15, 16, 19]. In this study, we further demonstrated that MERS VLPs could package the PS258(19712–19969)_ME_ RNA fragment of MERS-CoV, but not the SARS-CoV packaging signal PS580 (Fig. 3c). This suggests a specificity of the MERS-CoV RNA packaging. In addition, the involvement of the N protein in genome packaging may vary among the different coronaviruses. Previous studies demonstrate an N-dependent packaging of the SARS-CoV RNA packaging signal [19], but an N-independent packaging of the MHV RNA fragment was also suggested [24, 25]. In this study, an N-dependent packaging of MERS-CoV RNA was demonstrated (Fig. 3c).

The N protein of coronaviruses can be divided into two structural domains (NTD and CTD) and three intrinsically disordered regions (the N-arm, central linker region and C-tail) [10, 36]. Previous studies demonstrated the involvement of both the structural domains in binding the RNA of SARS-CoV and MHV (either independently or cooperatively as a bipartite) and showed the involvement of the C-terminal domain in oligomerization of the N protein [19, 30–34, 36–38]. In this study, the RNA-binding activity was demonstrated for the full-length N protein and both the N(1–263) and N(239–413) fragments that represent the NTD plus the flexible central linker region and the CTD, respectively (Fig. 5). The reduced RNA-binding activity of the N(1–156) fragment may indicate a critical role of the structural integrity of NTD. Alternatively, the central linker region may play a role in RNA binding. However, the N(264–413) fragment that lacks the N-terminal 25 amino acids of the N(239–413) fragment has an RNA-binding activity comparable to the N(239–413). This is different from the observation that the absence of the N-terminal 22 amino acids of SARS CoV N protein CTD significantly diminished its interaction with RNA [31]. Critical structures and sequences of the MERS-CoV N protein involved in RNA binding could be further studied. The MERS VLP system, which was established here for studying virus assembly and infection, could be used as a model system for evaluation of the efficacy of antiviral drugs.

## Conclusions

In this study, a MERS-CoV RNA packaging signal was identified by the detection of GFP expression following incubation of the MERS VLPs carrying the heterologous mRNA GFP-PS258(19712–19969)_ME_ with the virus permissive Huh 7 cells. Both the NTD and CTD of the MERS-CoV N protein showed binding activity to the 45-nt SL19805_ME_ RNA fragment that forms a stable stem-loop substructure of the viral RNA packaging signal PS258(19712–19969)_ME_. The MERS VLP system could help us understand virus infection and morphogenesis.
